# Double-mix pseudo-label framework: enhancing semi-supervised segmentation on category-imbalanced CT volumes

**DOI:** 10.1007/s11548-024-03281-1

**Published:** 2025-02-11

**Authors:** Luyang Zhang, Yuichiro Hayashi, Masahiro Oda, Kensaku Mori

**Affiliations:** 1 Graduate School of Informatics, Nagoya University, Furo-cho, Chikusa-ku, 464-8601 Nagoya, Aichi Japan; 2 Information Technology Center, Nagoya University, Furo-cho, Chikusa-ku, 464-8601 Nagoya, Aichi Japan; 3 Research Center of Medical Bigdata, National Institute of Informatics, 2-1-2 Hitotsubashi, Chiyoda-ku, 101-8430 Tokyo, Japan

**Keywords:** Semi-supervised Learning, CT Segmentation, Imbalanced Dataset

## Abstract

**Purpose:**

Deep-learning-based supervised CT segmentation relies on fully and densely labeled data, the labeling process of which is time-consuming. In this study, our proposed method aims to improve segmentation performance on CT volumes with limited annotated data by considering category-wise difficulties and distribution.

**Methods:**

We propose a novel confidence-difficulty weight (CDifW) allocation method that considers confidence levels, balancing the training across different categories, influencing the loss function and volume-mixing process for pseudo-label generation. Additionally, we introduce a novel Double-Mix Pseudo-label Framework (DMPF), which strategically selects categories for image blending based on the distribution of voxel-counts per category and the weight of segmentation difficulty. DMPF is designed to enhance the segmentation performance of categories that are challenging to segment.

**Result:**

Our approach was tested on two commonly used datasets: a Congenital Heart Disease (CHD) dataset and a Beyond-the-Cranial-Vault (BTCV) Abdomen dataset. Compared to the SOTA methods, our approach achieved an improvement of 5.1% and 7.0% in Dice score for the segmentation of difficult-to-segment categories on 5% of the labeled data in CHD and 40% of the labeled data in BTCV, respectively.

**Conclusion:**

Our method improves segmentation performance in difficult categories within CT volumes by category-wise weights and weight-based mixture augmentation. Our method was validated across multiple datasets and is significant for advancing semi-supervised segmentation tasks in health care. The code is available at https://github.com/MoriLabNU/Double-Mix.

## Introduction

Segmentation of organs and tissues from CT volumes is a crucial component of the computer-aided diagnosis paradigm, while the annotation process requires expert knowledge and is time-consuming. To address this issue, Semi-Supervised Learning (SSL), which utilizes both limited annotated and extensive unannotated data, has shown promising results in CT segmentation [1–3].

**Fig. 1:**
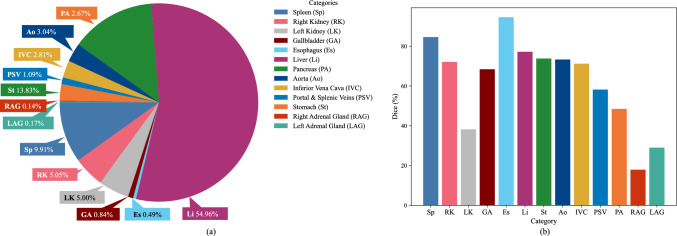
The category-wise voxel-count and Dice score of the fully-supervised segmentation on BTCV [2, 9] using V-Net [10]. **a** Voxel-counts, **b** Dice scores

In SSL, given the limited amount of labeled data, employing sample mixtures is widely used. CutMix [4] and ClassMix [5] enhance images by cutting and merging labeled and unlabeled images. CutMix utilizes annotated data, whereas ClassMix uses pseudo-labels to identify areas for merging. The category-wise voxel-count distribution in medical image segmentation is often highly imbalanced, as shown in Fig. 1a. The liver’s voxel-count is over 500 times higher than left-adrenal-gland, leading to a large difference in Dice score in full-supervision segmentation results in Fig. 1b. We defined these voxel-count differences as *distribution differences*. These mixture methods do not consider category-wise distribution imbalance, resulting in some categories with few voxels being easily overlooked during merging and diminishing segmentation performance for these categories.

**Fig. 2:**
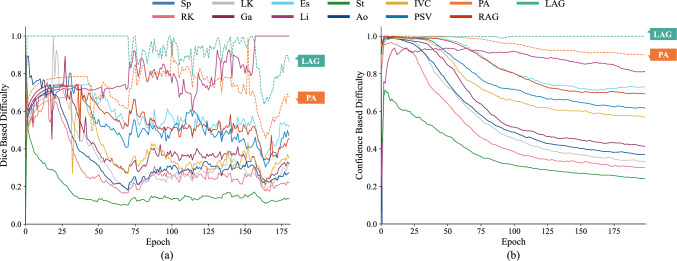
The category-wise difficulty evaluation during model training. a Based on Dice score [2], b based on confidence. The weights based on confidence are smoother than those based on Dice, which is particularly noticeable in small categories, such as PA (vibrant orange, dashed) and LAG (muted teal, dashed)

Another method for SSL is using the co-model-training framework such as cross-pseudo-supervision (CPS) [6], concurrently training two models to achieve similar outputs. It still faces category imbalance during training. Lin et al. [3] addressed category bias by applying category-wise distribution-based weight during co-model training based on CPS. However, they did not consider the heterogeneity of the two models in co-model training. Related work [7] shows that to achieve enhanced generalization capabilities, individual models should be both accurate and heterogeneous. As described in related work [2], it is necessary to increase the heterogeneity between the two models during co-model training. They compute difficulty weights based on Dice scores and voxel-count distribution weights for co-model training within the CPS framework, improving model performance by increasing the heterogeneity between the two models. However, when employing category imbalance datasets, the Dice score-based difficulty weight may have notable fluctuations during the training process, as illustrated in Fig. 2a. For some low voxel-count categories, even small pixel-level prediction errors can significantly impact the performance metrics for these small categories, leading to huge variations in the Dice score in the training process. This variability causes fluctuations in training, which affect the performance of the model. To address this issue, the confidence in the model’s output for each category, exhibiting the category-wise uncertainty, may provide a more stable metric for assessing the difficulty of each category [8]. As shown in Fig. 2b, confidence changes smoother during training, and the differences in evaluated difficulty among various categories become more pronounced. However, using only confidence as a measure for difficulty weights may not be sensitive to the categories with high changing speed in difficulty during the training process. Therefore, when estimating the category-wise difficulty, both category-wise confidence and Dice values should be taken into consideration.

In this work, we introduce a comprehensive approach to address category imbalance in semi-supervised CT volume segmentation. The primary contributions are reflected in the following aspects: (i) Introduction of confidence-difficulty weight (CDifW): We propose the CDifW, a novel category-wise difficulty weight that integrates both confidence and Dice score. We also incorporate the distribution weight (DisW), calculated from category-wise voxel-counts, to further refine our method. (ii) Novel Double-Mix Pseudo-label (DMP) Module: To tackle the limitations of existing data augmentation strategies, we introduce the DMP module, which uses these weights to focus augmentation efforts on high-difficulty categories. (iii) Innovative Double-Mix Pseudo-label Framework (DMPF): We propose the DMPF. This novel co-model training framework employs a CPS approach to enhance focus on distinct category-wise differences.

By applying the CDifW and DisW within both the DMP module and the overall model training, our method aims to improve the segmentation performance of imbalanced categories in CT volumes.

**Fig. 3:**
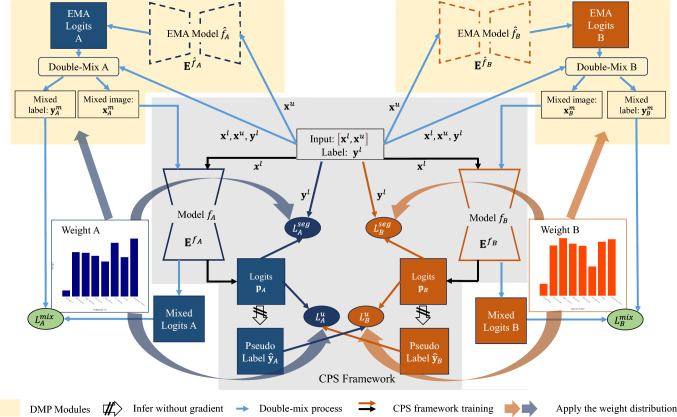
Overview of the proposed DMPF

**Fig. 4:**
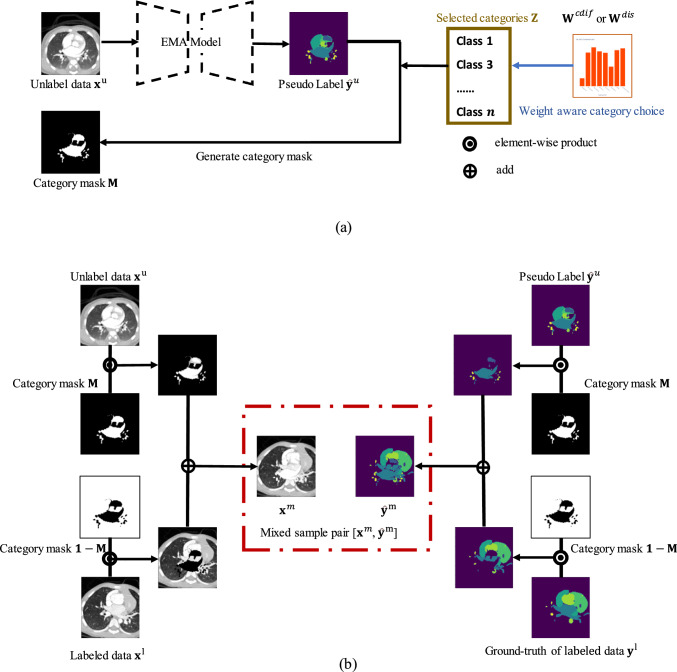
Overview of the proposed single DMP module. **a** Category mask $\textbf{M}$ generation. **b** Data Mixture

## Method

Our method, depicted in Fig. 3, integrates two DMP modules within a CPS-like training framework. The input for our method contain labeled $\textbf{x}^l$, unlabeled $\textbf{x}^u$, and the ground-truth $\textbf{y}^l$. The category-wise weights (in this work, CDifW and DisW) are used for loss calculation and category mask ${\textbf {M}}$ generation in the DMP module (see Fig. 4a). For segmentation models $f_A$ and $f_B$ in the CPS framework, we adopt Exponential Moving Average (EMA) [11] models $\hat{f}_A$ and $\hat{f}_B$ that have the same structure as the $f_A$ and $f_B$. The EMA models’ parameters $\textbf{E}^{\hat{f}_A}_t$ and $\textbf{E}^{\hat{f}_B}_t$ of $\hat{f}_A$ and $\hat{f}_B$ at iteration *t* are updated as $\textbf{E}^{\hat{f}_A}=\mu \textbf{E}_t^{f_A} + (1-\mu )\textbf{E}^{\hat{f}_A}_{t-1}$ and $\textbf{E}^{\hat{f}_B}=\mu \textbf{E}_t^{f_B} + (1-\mu )\textbf{E}^{\hat{f}_B}_{t-1}$ during training process, respectively. The DMP modules output mixed labels $\hat{\textbf{y}}^m_A$ and $\hat{\textbf{y}}^m_B$, defining new sample pairs for training $f_A$ and $f_B$. Framework details are described in Sect. “Double-mix Pseudo-label Framework”.

### Distribution based weight (DisW)

We utilized two weights, DisW and CDifW, to estimate the category-wise distribution and difficulty. $\textbf{x}^l$ and $\textbf{x}^u$ represent the labeled and unlabeled input volumes, respectively.

Following [2], we compute the category-wise distribution weight $w^{\textrm{dis}}_{t,k}$ for each category *k* at iteration *t* during training from the pseudo-labels $\hat{\textbf{y}}^u_t$ of $\textbf{x}^u$, by first calculating the voxel-count ratio $r_{t,k}$, and then normalizing these ratios using the category-wise voxel-counts $\psi _{t,k}^L$ based on the pseudo-labels $\hat{\textbf{y}}_t$ of the input $[\textbf{x}^l,\textbf{x}^u]$ by

1 $$\begin{aligned} w^{\textrm{dis}}_{t,k}  &   = \frac{\log (r_{t,k})}{\max _{\rho \in \{ 1,...,K\}} \log (r_{t,\rho })},\nonumber \\ r_{t,k}  &   = \frac{{\max _{\eta \in \{ 1,...,K\}} \psi _{t,\eta }^L}}{\psi ^L_{t,k}}. \end{aligned}$$

Weights are updated using an EMA approach

2 $$\begin{aligned} \textbf{W}^{\textrm{dis}}_t = \beta \textbf{W}^{\textrm{dis}}_{t-1} + (1 - \beta )\hat{\textbf{W}}^{\textrm{dis}}_t, \quad \hat{\textbf{W}}^{\textrm{dis}}_t=(w^{\textrm{dis}}_{t,1}, \ldots , w^{\textrm{dis}}_{t,K}), \end{aligned}$$

where $\beta$ is the parameter for weight smoothing, and *K* is the number of categories.

### Confidence-difficulty based weight (CDifW)

We assess the difficulty of each category through two dimensions: Dice score and confidence.

The learning speed is calculated by population stability index [12] based on the Dice score, which is defined as

3 $$\begin{aligned} d^u_{t,k}&= \sum _{\varrho =\partial }^{t} \mathbb {I}_{\Delta \le 0 }\ln \left( \frac{\zeta _{\varrho ,k}}{\zeta _{\varrho -1,k}}\right) , \nonumber \\ d^l_{t,k}&= \sum _{\varrho =\partial }^{t} \mathbb {I}_{\Delta > 0}\ln \left( \frac{\zeta _{\varrho ,k}}{\zeta _{\varrho -1,k}}\right) , \nonumber \\ \partial&=\max (t-\tau ,0), \end{aligned}$$

where $\zeta _{t,k}$ is the Dice score of the category *k* at iteration *t*, $\Delta = \zeta _{t,k}-\zeta _{t-1,k}$ and $\mathbb {I}_{\Delta \le 0 }$ and $\mathbb {I}_{\Delta > 0 }$ are defined as the indicator functions,

4 $$\begin{aligned} \mathbb {I}_{\Delta \le 0 } = {\left\{ \begin{array}{ll} 1 &  \text {if } \Delta \le 0 \\ 0 &  \text {if } \Delta> 0 \end{array}\right. }, \quad \mathbb {I}_{\Delta> 0} = {\left\{ \begin{array}{ll} 1 &  \text {if } \Delta > 0 \\ 0 &  \text {if } \Delta \le 0 \end{array}\right. }. \end{aligned}$$

The symbol $\tau$ represents the cumulative number of iterations, which is empirically set as 50. Following [2], $d^u_{t,k}$ and $d^l_{t,k}$ are used to evaluate whether the category *k* has been unlearned or well learned, and the difficulty $d_{t,k}$ should be defined as $d_{t,k} = (\frac{d^u_{t,k} + \epsilon }{d^l_{t,k} + \epsilon })^\alpha$, where $\epsilon$ is a smoothing element and the $\alpha$ is a hyperparameter to alleviate outliers. The well-learned category should perform low $d_{t,k}$. The difficulty weight for category *k* at iteration *t* is known as

5 $$\begin{aligned} w^{\textrm{dif}}_{t,k} = (1-\zeta _{t,k}) d_{t,k}. \end{aligned}$$

As mentioned in the Introduction, it is necessary to utilize confidence in category-wise difficulty. For labeled data $\textbf{x}^L$, the confidence $c_k$ for category *k* is computed from the logits $\textbf{p}^L$, where $\textbf{p}^L = \text {Softmax}\{f(\textbf{x}^L)\}$ and *f* is the segmentation model applying CDifW, $f\in \{f_A,f_B\}$. At iteration *t*, the category confidence $\hat{c}_{t,k}$ in a mini-batch is defined as

6 $$\begin{aligned} \hat{c}_{t,k} = \frac{1}{B}\sum _{b=1}^{B} \frac{1}{z_k}\sum _{j} p^L_{b,k,j}, \end{aligned}$$

where *B* is the mini-batch size and $p^L_{b,k,j}$ is the probability of category *k* at location *j* in sample *b*, with *j* indicating positions marked as category *k* in the ground-truth. The term $z_k$ represents the number of pixels for category *k* in the ground-truth $\textbf{y}$. The EMA method updates the confidence score $c_{t,k}$ as

7 $$\begin{aligned} c_{t,k} = \beta c_{t-1,k} + (1 - \beta ) \hat{c}_{t,k}. \end{aligned}$$

Similarly to [13], we define the information score $\textbf{S}_t$ for category *k* at iteration *t* as

8 $$\begin{aligned} \textbf{S}_{t}= &   \{\,s_{t,k} \mid k = 1, 2, \dots , K \,\},\nonumber \\ s_{t,k}= &   \frac{1-{c_{t,k}}}{\max _{\iota \in \{ 1,...,K\}} (1-c_{t,\iota })}. \end{aligned}$$

Then, our proposed CDifW $\textbf{W}^{\textrm{cdif}}$ in iteration *t* can be defined as

9 $$\begin{aligned} \textbf{W}^{\textrm{cdif}}_t=(w^{\textrm{cdif}}_{t,1} ,w^{\textrm{cdif}}_{t,2} \dots , w^{\textrm{cdif}}_{t,k} ), \quad w^{\textrm{cdif}}_{t,k} = s_{t,k}^{\gamma } w^{\textrm{dif}}_{t,k}, \end{aligned}$$

where the parameter $\gamma$ is a hyperparameter.

This method yields two sets of weights, $\textbf{W}^{\textrm{cdif}}$ and $\textbf{W}^{\textrm{dis}}$, representing training difficulty and category distribution, respectively. We omit subscript *t* for brevity.

### Double-mix pseudo-label module

The scarcity of labeled data necessitates using unlabeled data for augmentation. ClassMix [5] blends regions using pseudo-labels but does not rectify category imbalances, especially in high-difficulty categories, as described in the Introduction. Our DMP module counters this by applying weights $\textbf{W}^{\textrm{cdif}}$ and $\textbf{W}^{\textrm{dis}}$ from Sects. “Distribution based Weight (DisW)” and “Confidence-Difficulty based Weight (CDifW)” to selectively blending categories with ClassMix for more balanced and effective augmentation.

The process of a single DMP module is shown in Fig. 4. Initially, for an input unlabeled volume $$ \textbf{x}^u $$, its pseudo-label $$ \hat{\textbf{y}}^u $$ is computed using the EMA model. For data mixing, a binary mask should be created by the selected categories $$ \textbf{Z} $$. A probability distribution is generated using category-wise weights $$\textbf{W}$$ (in this work, $$\textbf{W} \in \{\textbf{W}^{\textrm{dis}}, \textbf{W}^{\textrm{cdif}}\}$$), where the weight $$w_k$$ for category $$k$$ represents the probability of this category being sampled. We sample $$k$$ times from this probability distribution, resulting in a set of selected categories denoted as $$\textbf{Z}$$. Using this categories set $$\textbf{Z}$$, we generate a binary mask $$ \textbf{M} $$ corresponding to an unlabeled volume $$ \textbf{x}^u $$ as follow: for any given pixel $$ j$$ in the volume, if $$\hat{\textbf{y}}^u_{j} \in \textbf{Z} $$, then $$ \textbf{M}_{j} = 1 $$; otherwise, $$ \textbf{M}_{j} = 0 $$. Therefore, the mixed sample pair $$[\textbf{x}^m,\hat{\textbf{y}}^m]$$ using unlabeled data $$\textbf{x}^u$$, labeled data $$\textbf{x}^l$$, and the ground-truth of $$\textbf{x}^l$$ can be obtained as

10 $$\begin{aligned} \textbf{x}^m = \textbf{x}^u \odot \textbf{M} + \textbf{x}^l \odot (\textbf{1}-\textbf{M}),\quad \hat{\textbf{y}}^m = \hat{\textbf{y}}^u \odot \textbf{M} + \textbf{y}^l \odot (\textbf{1}-\textbf{M}), \end{aligned}$$

where $\odot$ is an element-wise product. As shown in Fig. 3, during the training process, we employ distinct weight distributions to perform two DMP operations to obtain two different mixed sample pairs $[\textbf{x}^m_A, \textbf{y}^m_A]$ and $[\textbf{x}^m_B, \textbf{y}^m_B]$. This approach considers both the difficulty and distribution of each category, focusing on the imbalanced category augmentation.

### Double-mix pseudo-label framework

The process of DMPF is shown in Fig. 3. The updating process of $\textbf{W}^{\textrm{dis}}$ and $\textbf{W}^{\textrm{cdif}}$, as well as the generation process of our proposed DMP, can be summarized in Algorithm 1. To simultaneously consider the distribution and difficulty of categories, we created two models, $f_A$ and $f_B$, with different random initializations for the model weights. As we defined in Sect. “Distribution based Weight (DisW)”, $N^L$ and $N^U$ show the sample number of the labeled dataset and the unlabeled dataset. We obtained logits $\textbf{p}_A$ and $\textbf{p}_B$ of the input data $[\textbf{x}^l,\textbf{x}^u]$ through $f_A$ and $f_B$. In the CPS framework, the supervised loss is

11 $$\begin{aligned} L^{s}_{\textrm{seg}}(\textbf{p}_A,\textbf{p}_B,\textbf{y})&= \frac{1}{N^L} \frac{1}{K} \sum _{i=0}^{N^L} [ L_{s}( \textbf{W}^{\textrm{cdif}}, \textbf{p}_{A_i}, \textbf{y}_i) \nonumber \\&\quad + L_{s}(\textbf{W}^{\textrm{dis}}, \textbf{p}_{B_i}, \textbf{y}_i)], \end{aligned}$$

where $\textbf{y}$ is the ground-truth of labeled data $\textbf{x}^l$, and for the unsupervised loss component

12 $$\begin{aligned} L^{s}_u(\textbf{p}_A,\textbf{p}_B, \hat{\textbf{y}}_A, \hat{\textbf{y}}_B)&= \frac{1}{N^L + N^U} \frac{1}{K} \sum _{q=0}^{N^L+N^U} [ L_u(\textbf{W}^{\textrm{cdif}}, \textbf{p}_{A_q}, \hat{\textbf{y}}_{B_q}) \nonumber \\&\quad + L_u(\textbf{W}^{\textrm{dis}}, \textbf{p}_{B_q}, \hat{\textbf{y}}_{A_q})], \end{aligned}$$

where $\hat{\textbf{y}}_A$, $\hat{\textbf{y}}_B$ are the pseudo-labels calculated from $\textbf{p}_A$ and $\textbf{p}_B$. In our experiments, $L_{s}(\textbf{W}, \textbf{x}, \textbf{y}) = L_{\text {Dice}}( \textbf{W}, \textbf{x}, \textbf{y}) + \frac{1}{2} L_{\text {CE}}(\textbf{W}, \textbf{x}, \textbf{y})$ and $L_u(\textbf{W}, \textbf{x},\textbf{y}) = L_{\text {CE}}( \textbf{W}, \textbf{x}, \textbf{y})$, where $L_{\text {CE}}$ was set as the weighted cross-entropy loss and $L_{\text {Dice}}$ was set as the weighted Dice loss [14].

**Algorithm 1:**
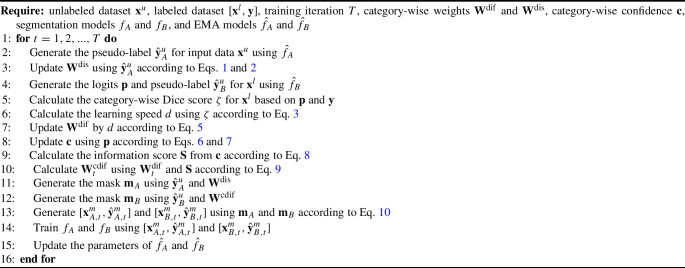
Double-Mix Pseudo-Label Framework

In the DMP module, $\hat{\textbf{y}}^u_A$ and $\hat{\textbf{y}}^u_B$ are the pseudo-labels generated by the unlabeled volumes $\textbf{x}^u$ from the EMA model $\hat{f}_A$ and $\hat{f}_B$. $[\textbf{x}^u,\hat{\textbf{y}}^u_A,\textbf{x}^l,\textbf{y}]$ and $[\textbf{x}^u,\hat{\textbf{y}}^u_B,\textbf{x}^l,\textbf{y}]$ are, respectively, fed into two DMP modules which selecting categories by $\textbf{W}^{\textrm{cdif}}$ and $\textbf{W}^{\textrm{dis}}$. This process is employed to generate new training data pairs at each iteration, denoted as $[\textbf{x}^m_A,\hat{\textbf{y}}^m_A]$ and $[\textbf{x}^m_B,\hat{\textbf{y}}^m_B]$. The loss for the data pairs created by the DMP modules is

13 $$\begin{aligned} L^{m}_{\textrm{seg}}(\textbf{p}_A^m,\textbf{p}_B^m,\hat{\textbf{y}}^m_A,\hat{\textbf{y}}^m_B) = L_{s}(\textbf{W}^{\textrm{cdif}},\textbf{p}_A^m,\hat{\textbf{y}}^m_A) +L_{s} (\textbf{W}^{\textrm{dis}}, \textbf{p}_B^m,\hat{\textbf{y}}^m_B), \end{aligned}$$

where $\textbf{p}_A^m$ and $\textbf{p}_B^m$ are the output of $f_A$ and model $f_B$ with input $\textbf{x}_A^m$ and $\hat{\textbf{x}}_B^m$. Therefore, the loss function can be defined as

14 $$\begin{aligned} L  &   = L^{s}_{\textrm{seg}}(\textbf{p}_A,\textbf{p}_B,\textbf{y}) + L^m_{\textrm{seg}}(\textbf{p}_A^m,\textbf{p}_B^m,\hat{\textbf{y}}^m_A,\hat{\textbf{y}}^m_B) \nonumber \\  &   \quad \ + \theta L^{s}_u(\textbf{p}_A, \textbf{p}_B, \hat{\textbf{y}}_A, \hat{\textbf{y}}_B), \end{aligned}$$

where $\theta$ is a hyperparameters, and the epoch-dependent Gaussian ramp-up strategy [3] is used to enlarge the ratio of unsupervised loss.

In inference stage, for the input volume $\textbf{x}^p$, we calculate $\textbf{p}^p_A = f_A(\textbf{x}^p)$ and $\textbf{p}^p_B = f_B(\textbf{x}^p)$. The predicted logits are given by $\textbf{p}^p = \frac{ (\textbf{p}^p_A + \textbf{p}^p_B)}{2}$. The predicted result $\textbf{y}^p$ is derived from $\textbf{p}^p$ by assigning each voxel to the category with the highest predicted probability.

**Table 1:** Segmentation outcomes between our method and other SSL segmentation methods on 40% labeled BTCV dataset

Methods	Average Dice and ASD		Average Dice (%) of Each Class $\uparrow$												
	Dice (%) $\uparrow$	ASD $\downarrow$	Sp	RK	LK	Ga	Es	Li	St	Ao	IVC	PSV	PA	RAG	LAG
SS-Net [1]${*}$	42.5 ± 6.5	49.2 ± 10.1	84.9	82.1	77.1	10.1	0.0	90.2	51.0	78.2	62.0	0.0	17.2	0.0	0.0
DST [18]${*}$	40.1 ± 0.9	46.8 ± 2.2	84.9	78.5	73.4	4.5	0.0	**91**.**4**	49.9	72.7	52.1	1.0	12.6	0.0	0.0
Depl [19]${*}$	41.2 ± 0.9	48.1 ± 0.5	82.1	81.7	74.2	3.3	0.0	90.8	51.9	74.4	57.1	0.0	19.7	0.0	0.0
CPS [6]${*}$	37.5 ± 2.1	52.5 ± 11.1	85.7	77.9	71.2	4.4	0.0	91.3	48.1	66.9	31.8	0.0	10.2	0.0	0.0
CReST [20]${*}$	38.5 ± 3.8	22.1 ± 8.7	62.6	67.5	68.9	18.2	21.2	81.7	42.9	53.1	35.7	6.6	14.0	18.1	9.5
CLD [3]${*}$	54.7 ± 1.2	7.6 ± 0.6	** 88.0**	84.6	80.3	23.0	28.7	90.4	51.5	79.2	65.1	42.8	24.7	25.3	27.0
DHC [2]${*}$	59.6 ± 1.2	4.5 ± 0.6	87.2	84.1	79.6	**40**.**3**	44.8	83.4	53.6	80.0	66.6	**46**.**5**	35.0	33.1	40.9
Ours w/o DMP	60.0 ± 0.7	**3**.**9** ± **0**.**5**	84.6	85.5	80.0	32.6	45.8	86.6	**56**.**2**	79.6	66.8	45.7	**37**.**5**	28.9	**50**.**5**
Ours	**61**.**2** ± **0**.**7**	4.06 ± 0.6	86.9	**86**.**0**	**82**.**3**	35.3	**48**.**5**	86.5	53.1	**81**.**1**	**71**.**0**	44.8	35.4	**36**.**4**	48.0

The best performance is denoted using bold. The second-best performance is marked by underline. The results are presented in the form of “mean ± std.”

${*}$ We implement methods on our dataset

**Table 2:** Segmentation outcomes between our method and other SSL segmentation methods on 5% labeled CHD dataset

Methods	Average Dice and ASD		Average Dice (%) of Each Class $\uparrow$						
	Dice (%) $\uparrow$	ASD $\downarrow$	LV	RV	LA	RA	My	Ao	PA
SS-Net [1]${*}$	49.7 ± 7.2	7.9 ± 0.5	58.4	48.7	50.5	53.3	59.7	33.6	43.5
DST [18]${*}$	62.3 ± 1.4	5.6 ± 0.5	70.3	66.1	68.8	65.3	**67**.**2**	48.2	49.8
Depl [19]${*}$	63.6 ± 3.8	**5**.**1** ± **0**.**3**	71.9	66.8	72.3	67.9	66.9	49.7	49.6
CPS [6]${*}$	62.0 ± 0.9	5.5 ± 0.3	71.2	67.2	69.2	63.9	66.9	48.3	47.2
CReST [20]${*}$	61.5 ± 1.5	6.4 ± 0.9	68.9	64.6	71.3	63.3	65.8	45.7	50.9
CLD [3]${*}$	62.4 ± 1.6	5.9 ± 0.5	68.6	64.3	72.8	66.3	66.1	47.0	51.5
DHC [2]${*}$	64.1 ± 0.2	6.7 ± 0.5	70.9	68.4	72.6	**70**.**9**	66.0	49.2	50.9
Ours w/o DMP	64.3 ± 1.3	6.0 ± 0.6	72.4	68.9	72.9	69.7	65.4	49.3	51.6
Ours	**66**.**5** ± **0**.**1**	6.0 ± 0.4	**73**.**7**	**72**.**1**	**77**.**3**	68.6	60.5	**56**.**9**	**56**.**0**

$^{*}$ We implement methods on our dataset

$^{*}$ The best performance is denoted using bold. The second-best performance is marked by underline. The results are presented in the form of "mean ± std"

**Fig. 5:**
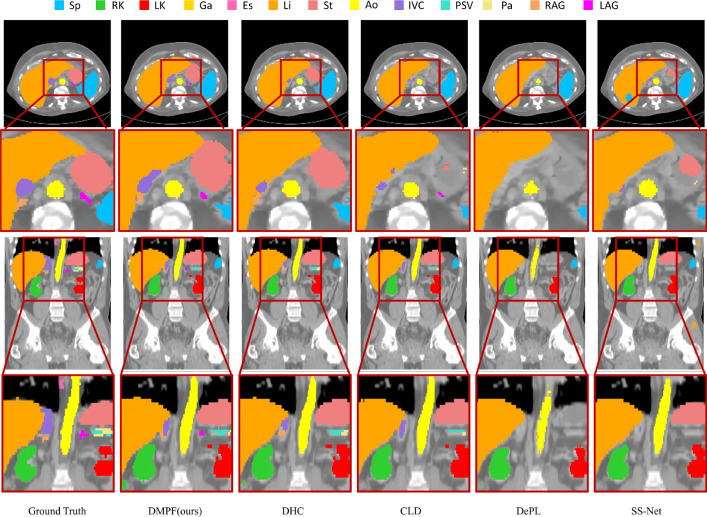
Comparative experiments with other methods using 40% BTCV dataset. Some high-difficulty categories are highlighted by red frames

**Fig. 6:**
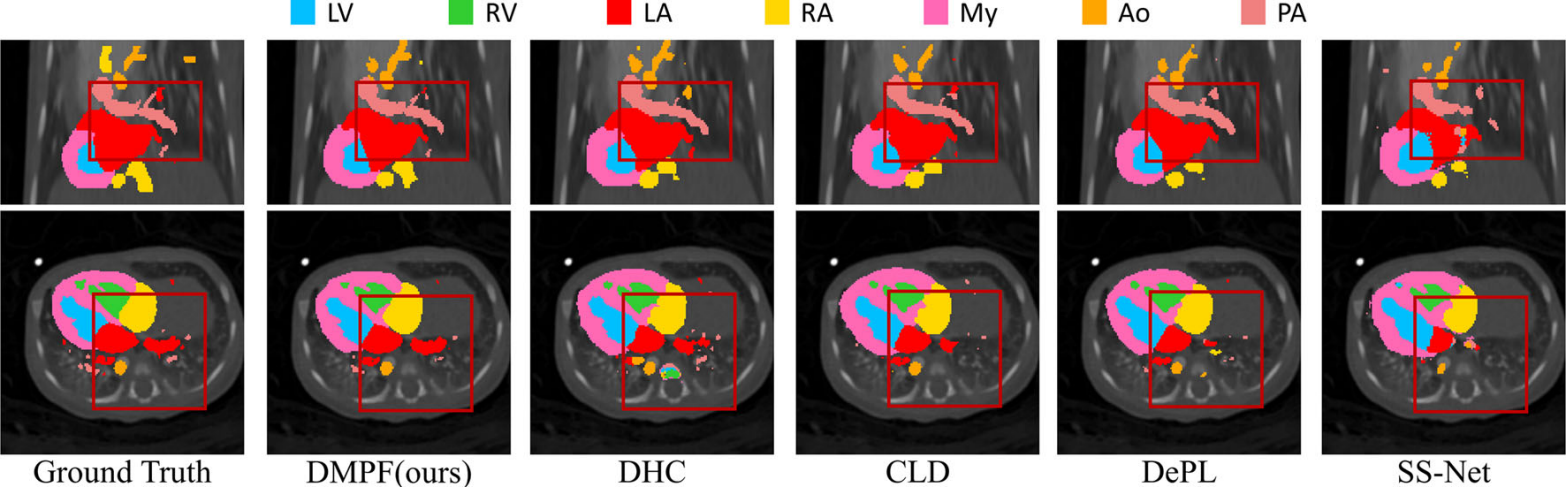
Comparative experiments with other methods using 5% of the CHD dataset

## Experiment

### Dataset

In this experiment, we validate our approach using two public imbalanced datasets: the BTCV dataset [9] for abdominal organ segmentation, known for its extreme category imbalance, containing 30 annotated cases. For the total cases, 4 were used for validation, 6 for testing, and the others for training. The CHD dataset for Congenital Heart Disease [15] is notable for its categories with balanced voxel-counts and imbalanced segmentation difficulty. The CHD dataset contains 110 CT volumes with labels. Elevan cases were used as the test set, another 11 as the validation set, and the others for the training set. We also tested the effectiveness of DMPF on the balanced dataset, the WRAMC [16], which contains 116 colon cases without annotations, among which we annotated 10 cases, containing the categories of air-area and solid-material, to test the performance of our model on relatively balanced and simpler tasks.

### Experimental settings

In our experiment, for the BTCV dataset, we used 10%, 20%, and 40% of the training set as labeled data; for CHD, 5%, 10%, and 20% of the training set as labeled data. For the WRAMC, we applied three-fold cross-validation on ten annotated cases. Unannotated data served as unlabeled training data. We used a patch size of (128, 128, 64) voxels by representing width, height, and depth, respectively. The final segmentation results were obtained using a sliding window strategy with a stride size of (32, 32, 16). We utilized a CPS-like framework with two 5-layer V-Net [10] with kernel numbers of [32, 64, 128, 256, 512] for each layer in the encoder and decoder as the baseline for segmentation. The co-model framework involved two randomly initialized models using the Kaiming normal method [17]. We used random-flip and random-crop to all volumes as the augmentation and used a poly-decay learning strategy [14] with an initial rate of 0.03, optimized via SGD with 0.9 momentum. Hyperparameters were set empirically: $\alpha$ at 0.5, $\beta$ at 0.99, $\gamma$ at 0.2, and $\theta$ at 0.1. Training employed an early stopping strategy with a 30-epoch threshold. We applied two widely used metrics, Dice score and Average Surface Distance (ASD), in our study.

## Results and discussion

### Result analysis

In our experiments, we conducted comparisons with several SOTA semi-supervised segmentation methods [1, 6, 18, 19]. Additionally, we adapted category imbalance strategies [2, 3, 20] to the CPS for segmentation tasks, benchmarking them against our approach. We used different initialization seeds for three trials in each experiment. Identical seeds ensure consistent initial parameters across models. “ours w/o DMP” shows the result using the CDifW and DisW without DMP module, while “ours” shows the result using our proposed DMPF.

**Fig. 7:**
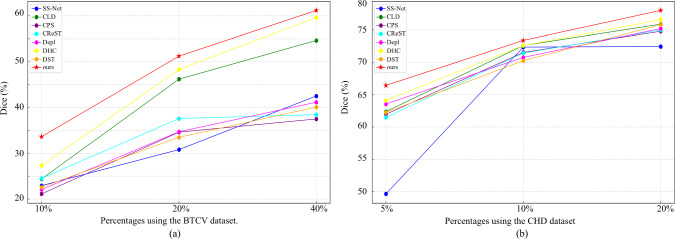
Results using different percentages of labeled data. **a** BTCV, **b** CHD

**Fig. 8:**
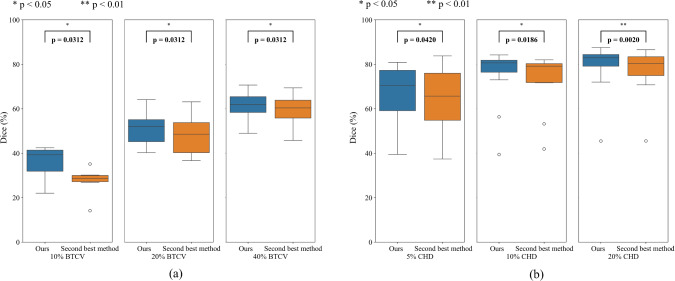
The result of Wilcoxon signed-rank test. **a** The results using 10%, 20%, and 40% of the BTCV dataset as the labeled data, **b** the results using 5%, 10%, and 20% of the CHD dataset as the labeled data

Figure 1 reveals the BTCV dataset’s imbalanced categories with low voxel-count in Es, RAG, and LAG. As demonstrated in the “ours” of Table 1, our approach improved the Dice score by an average of 4.7% in this imbalanced category than the baseline. Our method also enhanced the Dice score by an average of 5.0% in imbalanced high-difficulty categories RV, Ao, and PA in CHD dataset than the baseline, as shown in Table 2. Figures 5 and 6 show the segmentation results on BTCV and CHD. Our methods performed better than other related methods.

**Table 3:** The results of training CPS module using different weights on 10% labeled BTCV dataset

Methods	Average Dice and ASD	
	Dice (%) $\uparrow$	ASD $\downarrow$
DHC [2]	29.8 ± 5.4	28.1 ± 8.5
CDifW-DisW	**29.9** ± **2**.**7**	25.3 ± 8.3
CDifW-CDifW	26.4 ± 2.8	**25**.**2** ± **3**.**5**
CDifW-CDisW	29.4 ± 2.6	27.3 ± 3.4
DisW-DisW	26.21 ± 4.7	32.2 ± 5.0
CDisW-CDisW	28.9 ± 2.6	25.5 ± 4.4

$^{*}$ The best performance is denoted using bold. The second-best performance is marked by underline. The results are presented in the form of "mean ± std"

**Table 4:** Comparison with other data mixture augmentations on 10% labeled BTCV dataset

Methods	Average Dice and ASD	
	Dice (%) $\uparrow$	ASD $\downarrow$
CDifW-DisW	29.9 ± 2.7	25.3 ± 8.3
CutMix [4]	31.5 ± 2.6	20.4 ± 5.8
CutOut [21]	30.9 ± 3.7	24.5 ± 6.8
ClassMix [5]	29.3 ± 8.3	33.1 ± 7.0
Ours	**35**.**7** ± **1**.**0**	**18**.**2** ± **4**.**3**

$^{*}$ The best performance is denoted using bold. The second-best performance is marked by underline. The results are presented in the form of "mean ± std"

**Fig. 9:**
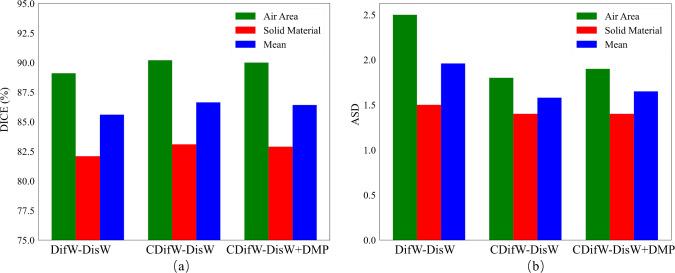
Dice and ASD results from three-fold cross-validation on colon dataset. **a** Dice scores, **b** ASDs

**Fig. 10:**
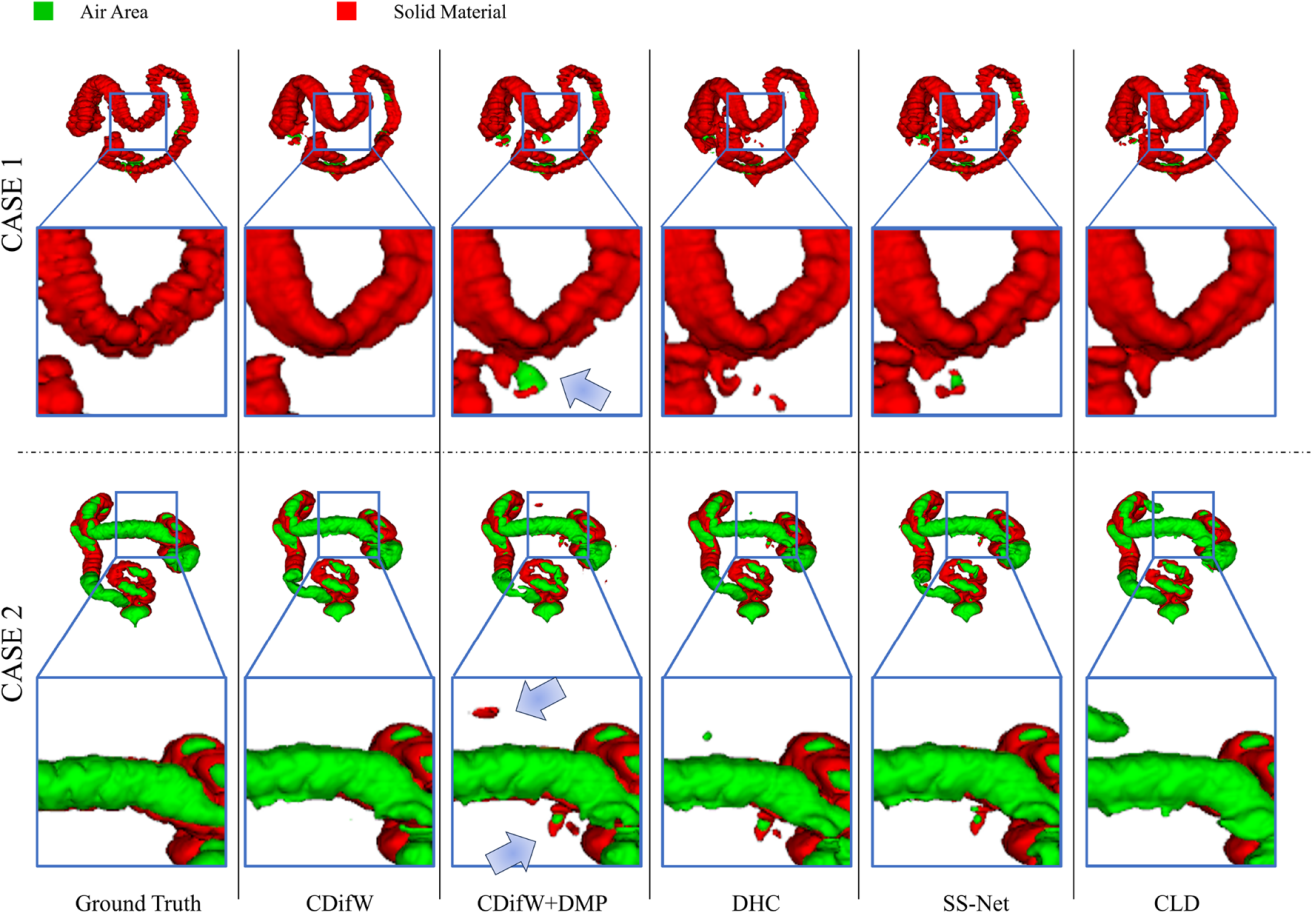
Segmentation results on balanced colon segmentation dataset. The arrow indicates a potential erroneous segmentation likely due to the disruption of spatial information by ClassMix

Furthermore, our proposed CDifW improved the Dice score by an average of 3.0% in the imbalanced categories on BTCV, and 0.4% in the imbalanced categories on CHD, as shown in the “ours w/o DMP” in Tables 1 and 2.

Our method boosted segmentation accuracy in these challenging categories, as evidenced by the average Dice score improvements of 2.3% and 1.5% than the baseline in the 5% CHD and 40% BTCV labeled datasets, respectively. This was achieved through targeted data augmentation, considering category-wise confidence and distribution. The method’s effectiveness on different percentages is shown in Fig. 7. Experimental results indicated significant improvement in segmentation performance considering category-wise confidence. Subsequently, employing the DMP module for data augmentation in high-difficulty categories effectively enhanced overall model performance. Additionally, our method demonstrated more pronounced effectiveness when working with low percentages of labeled data, as shown in Fig. 7.

We conducted Wilcoxon signed-rank tests on the average Dice scores for each sample, comparing the results across different dataset splits and experimental conditions. Figure 8a shows the results using 10%, 20%, and 40% of the BTCV dataset as the labeled data, while Fig. 8b illustrates the results using 5%, 10%, and 20% of the CHD dataset as the labeled data. In all experiments, the Dice scores of our method were statistically higher than those of the second-best method, with all p-values being less than 0.05 ($p < 0.05$). These results confirm the superiority of our method across different levels of labeled data availability.

### Ablation studies

We conducted two ablation studies to examine: (1) the effect of using two distinct category-wise weights in model training, and (2) a comparison with other sample mixture methods.

In Table 3, we present comparative experiments with different weight combinations. For instance, "CDifW-DisW" used CDifW and DisW weights, respectively, during CPS model training. Additionally, CDisW is a weight derived by applying confidence score in Eq. (6) to DisW in Eq. (1), which consequently paid attention to both category-wise difficulty and distribution to a certain extent.

When the weights used in both models were similar (e.g., DisW-DisW, CDifW-CDifW), the models failed to comprehensively consider the category-wise differences in difficulty or distribution, leading to a decline in performance. Meanwhile, CDisW, by considering both category-wise difficulty and distribution, can improve model performance (as in CDisW-CDisW). However, combining CDifW-CDisW reduces heterogeneity due to applying confidence on DistW, causing lower performance. We hypothesized that high heterogeneity weight pairs can increase the model performance, and our proposed CDifW-DisW may provide higher heterogeneity than other methods.

Table 4 presents the results of training our method compared to other data augmentation techniques on 10% of the BTCV dataset. All the methods were based on a CDifW-DisW-applied CPS framework. The results demonstrate that the DMP module, which focuses on category-wise differences for data augmentation, performed best in the imbalanced dataset.

### Discussion on balanced dataset

We evaluated our method within a balanced colon segmentation dataset. The DifW was described in Eq. (3). As illustrated in Figs. 9 and 10, our “CDifW-DisW” focusing on category confidence enhanced the segmentation performance for each category. However, a notable limitation is that applying the DMP module (“CDifW-DisW+DMP”) to the balanced dataset likely compromises some spatial information, resulting in reduced performance, as shown in Fig. 10.

## Conclusion

This study presents the development and validation of the category-wise CDifW and DMP modules to enhance segmentation performance on imbalanced medical data. The CDifW module considers segmentation difficulty weights based on category-wise confidence, smoothing the weight during training, both improving segmentation in the imbalanced dataset and showing notable benefits in simpler tasks. The DMP module complements CDifW by augmenting the samples of difficult categories using a novel combination of actual and pseudo-labels, which surpasses other data mixture augmentation methods in performance. While our method notably improves performance, especially in high-difficulty categories and imbalanced images, the DMP module introduces segmentation noise in simpler tasks, as illustrated in Fig. 10. Future research will aim to reduce segmentation noise in DMPF by considering marginal contextual information and improve imbalance handling by refining the CDifW.

## Data Availability

The code is available at the following link: https://github.com/MoriLabNU/Double-Mix.

## References

[1] Wu Y, Wu Z, Wu Q, Ge Z, Cai J (2022) Exploring smoothness and class-separation for semi-supervised medical image segmentation. MICCAI, LNCS 13435:34–43

[2] Wang H, Li X (2022) DHC: Dual-debiased heterogeneous co-training framework for class-imbalanced semi-supervised medical image segmentation. MICCAI, LNCS 14222:582–591

[3] Lin Y, Yao H, Li Z, Zheng G, Li X (2022) Calibrating label distribution for class-imbalanced barely-supervised knee segmentation. MICCAI, LNCS 13438:109–118

[4] French G, Laine S, Aila T, Mackiewicz M, Finlayson G (2020) Semi-supervised semantic segmentation needs strong, varied perturbations. In: BMVC

[5] Olsson V, Tranheden W, Pinto J, Svensson L (2021) Classmix: Segmentation-based data augmentation for semi-supervised learning. WACV 1369–1378

[6] Chen X, Yuan Y, Zeng G, Wang J (2021) Semi-supervised semantic segmentation with cross pseudo supervision. CVPR 2613–2622

[7] Krogh A, Vedelsby J (1994) Neural network ensembles, cross validation, and active learning. NeurIPS 7:231–238

[8] Qiu J, Hayashi Y, Oda M, Kitasaka T, Mori K (2023) Class-wise confidence-aware active learning for laparoscopic images segmentation. Int J Comput Assist Radiol Surg 18(3):473–482 36271215 10.1007/s11548-022-02773-2

[9] Landman B, Xu Z, Igelsias J, Styner M, Langerak T, Klein A (2015) 2015 MICCAI Multi-Atlas Labeling Beyond Cranial Vault–Workshop Challenge

[10] Milletari F, Navab N, Ahmadi S-A (2016) V-Net: Fully convolutional neural networks for volumetric medical image segmentation. In: 3DV, pp. 565–571. IEEE

[11] Brown RG (1956) Exponential Smoothing for Predicting Demand. Little, Brown and Company, Boston

[12] Yurdakul B (2018) Statistical properties of population stability index (psi). PhD thesis, Western Michigan University

[13] Gal Y, Islam R, Ghahramani Z (2017) Deep bayesian active learning with image data. In: ICML, pp. 1183–1192. PMLR

[14] Isensee F, Jaeger PF, Kohl SA, Petersen J, Maier-Hein KH (2021) nnU-Net: a self-configuring method for deep learning-based biomedical image segmentation. Nat Methods 18(2):203–211 10.1038/s41592-020-01008-z 33288961

[15] Xu X, Wang T, Shi Y, Yuan H, Jia Q, Huang M, Zhuang J (2019) Whole heart and great vessel segmentation in congenital heart disease using deep neural networks and graph matching. MICCAI, Proceedings, Part II, LNIP 11765:477–485 Springer

[16] Long JR, Frew MI, Brazaitis MP (2011) Virtual colonoscopy in the US army: current utilization at the Walter Reed Army Medical Center. Abdom Imaging 36:149–152 20473668 10.1007/s00261-010-9623-7

[17] He K, Zhang X, Ren S, Sun J (2015) Delving deep into rectifiers: Surpassing human-level performance on imagenet classification. In: ICCV, pp. 1026–1034

[18] Chen B, Jiang J, Wang X, Wan P, Wang J, Long M (2022) Debiased self-training for semi-supervised learning. NeurIPS 35:32424–32437

[19] Wang X, Wu Z, Lian L, Yu SX (2022) Debiased learning from naturally imbalanced pseudo-labels. In: CVPR, pp. 14647–14657

[20] Wei C, Sohn K, Mellina C, Yuille A, Yang F (2021) CReST: A class-rebalancing self-training framework for imbalanced semi-supervised learning. In: CVPR, pp. 10857–10866

[21] Devries T, Taylor GW (2017) Improved regularization of convolutional neural networks with Cutout. CoRR arXiv:1708.04552

